# Death of Dr. Clement R. Harris

**Published:** 1871-11

**Authors:** 


### DEATH OF DR. CLEMENT R. HARRIS.

We sorrowfully record the death of one of our most esteemed and talented Associate Editors, Dr. Clement R. Harris, of Staunton, Virginia. We were painfully surprised when the sad intelligence reached us, it came so unexpected, so sudden. But a short time had elapsed, when, in health, and with a heart bounding with love for his profession which he so honored, he had communicated to us his views in reference to ennobling and elevating the calling to which he had so long and faithfully given the richest treasures of his heart and brilliant mind.

Dr. Harris was a native of Virginia, of the Old School, a gentleman endowed with all the noble characteristics so peculiar to the "Auld Lang Syne" of the Old Dominion.

Dr. Harris was a man of culture—of rare discrimination, and possessed of unusal force of character and intellectual power. Endowed with great versatility of talent, he for many years, represented his county in the Senate of Virginia, and as a debater, was recognized as worthy to take front-rank with the first men of his native State. His scientific acquirements were varied and extensive, and were universally aknowledged by those who shared the pleasure of his acquaintance. A few years ago he came within one vote of being elected one of the Professors of the University of Virginia, an institution of learning second to none in this country.

As a physician our lamented associate was a bright and a shining light. As a citizen he was generous, kind and affable—ever ready to tender the hand of benevlence to the afflicted, and ever the first to throw the mantle of charity over the faults of friends or enemies. In his social relations he was affectionate as husband, father or friend, warmhearted, unselfish and true. He was the eldest of a family of four brothers high in the love and esteem of their fellow-citizens. Hon. John J. Harris represents the Old Dominion, in part, in the Congress of the U. S. Rev. W. A. Harris is president of the Wesleyan Female Institute of Staunton. Both these gentlemen are widely known and esteemed as men of superior talents and moral worth. Dr. Harris was also a cousin of the late lamented Baptist scholar and divine—a pure and noble Georgian—Rev. N. M. Crawford, D. D., son of one of Georgia's brightest intellects of the past, Hon. W. H. Crawford.

To the profession of Virginia we tender our sad condolence in this great loss, and with the bereaved family we mourn over the fall of one dear to them, and beloved by all who knew him.

